# Deep Brain Stimulation for Dystonia in the Netherlands: A Delphi Study to Develop National Consensus

**DOI:** 10.5334/tohm.1070

**Published:** 2025-09-26

**Authors:** Liesanne M. Centen, Linda Ackermans, Annemieke I. Buizer, M. Fiorella Contarino, Joke M. Dijk, Annelien A. Duits, Carel Hoffmann, Mark L. Kuijf, Irene Kusnadi, D. L. Marinus Oterdoom, Laura A. van de Pol, P. Rick Schuurman, Marina A. J. Tijssen, Martje E. van Egmond

**Affiliations:** 1 Department of Neurology, University of Groningen, University Medical Center Groningen, Groningen, The Netherlands; 2 Expertise Center Movement Disorders Groningen, University of Groningen, University Medical Centre Groningen, Groningen, The Netherlands; 3 Department of Neurosurgery, Maastricht University Medical Center, Maastricht, The Netherlands; 4 Department of Rehabilitation Medicine, Amsterdam Movement Sciences and Emma Children’s Hospital, Amsterdam UMC, Amsterdam, The Netherlands; 5 Department of Neurology, Haga Teaching Hospital, The Hague, The Netherlands; 6 Department of Neurology, Leiden University Medical Center, Leiden, The Netherlands; 7 Department of Neurology, Academic Medical Center Amsterdam, Amsterdam, The Netherlands; 8 Department of Medical Psychology, Maastricht & Radboud University Medical Centre, Maastricht & Nijmegen, The Netherlands; 9 Department of Neurosurgery, Haga Teaching Hospital, The Hague, The Netherlands; 10 Department of Neurology, Maastricht University Medical Center, Maastricht, The Netherlands; 11 Department of Neurosurgery, University of Groningen, University Medical Center Groningen, Groningen, The Netherlands; 12 Department of Child Neurology, Amsterdam Neuroscience and Emma’s Children’s Hospital, Amsterdam University Medical Center, Amsterdam, The Netherlands; 13 Department of Neurosurgery, Academic Medical Center Amsterdam, Amsterdam, The Netherlands

**Keywords:** Dystonia, treatment, deep brain stimulation, Delphi study

## Abstract

**Background:**

Predicting outcome for individuals with dystonia undergoing treatment with deep brain stimulation (DBS) remains challenging. This is further complicated by a lack of uniform screening, follow-up, and heterogeneous outcome measures. This study aims to achieve consensus on a national level among experts in the field to develop an agreed set of outcome measures and introduce more uniformity in the process of preoperative screening and follow-up.

**Methods:**

A modified Delphi process was conducted among experts in the field of DBS for dystonia. The process consisted of an inventory round, followed by two rounds of Delphi questionnaires, closing with a digital consensus meeting. Experts rated the importance of items within several categories: ((non-)motor symptoms, selection criteria, follow-up, DBS-related aspects, involved care providers). A threshold of 70% was maintained as consensus criterium.

**Results:**

After the first two rounds, consensus was reached on 40/59 items (adult DBS), and 47/61 items (pediatric DBS). The remaining items were rephrased into 28 statements (13 adult DBS, 13 pediatric DBS, and 2 concerning both) and voted on during a final consensus meeting. There, 23/28 statements (11 adult, 11 pediatric, 1 both) reached consensus. Overall, after three rounds, on most items consensus was reached.

**Discussion:**

In this Delphi study, a high level of consensus among national experts was achieved on outcome measures and the process of screening and follow-up in DBS for dystonia for adults and children. The results present national consensus and offer an excellent start for collaborative international studies on best practice for DBS in dystonia.

## Introduction

Dystonia is a hyperkinetic movement disorder defined by continuous muscle contractions that lead to involuntary movements and postures or both [1]. First line medical treatment consists of botulinum toxin injections and medication. For medication-refractory dystonia cases, Deep Brain Stimulation (DBS) is available as a more advanced treatment option. The most commonly used electrode target is the globus pallidus interna (GPi), with the subthalamic nucleus (STN) having emerged more recently as an alternative option. Treatment with DBS can be very effective in controlling motor symptoms of dystonia in subgroups of patients, with several trials substantiating its efficacy in both short and long-term follow-up [2,3]. Still, to this day it remains difficult to predict beforehand whether patients will experience a beneficial effect. Reported non-responder rates in literature are as high as 25% [2].

Several factors are known to influence the success of DBS treatment. On the one side there are DBS-related factors such as electrode placement, stimulation settings, and chosen target, while on the other side are dystonia-related factors such as disease duration, underlying etiology for the dystonia, and body distribution [4]. For instance, patients with inherited (e.g. DYT1) or idiopathic dystonia, tend to show better results compared to patients with acquired dystonia [5]. The underlying rationale is that brain network damage in acquired forms reduces the efficacy of neuromodulation [4]. Moreover, in terms of body distribution, bulbar or laryngeal dystonia tends to respond less well to DBS compared to dystonia affecting the limbs, neck or trunk [6,7]. With regard to DBS-related factors, as mentioned previously, while the globus pallidus interna (GPi) remains the most well-studied and commonly used target, some studies explored the subthalamic nucleus (STN) as an alternative with promising results [8]. In addition, electrode placement and stimulation settings are closely linked and jointly determine the effectiveness of DBS. Finally, for dystonia the benefit from DBS is not instantaneous and takes months to years to develop, further complicating outcome prediction. A lot remains unknown about the interplay between DBS-related and dystonia-related factors. All in all, several research gaps remain, among which are the need for a better understanding of factors contributing to reduced or non-response of DBS, further optimization of stimulation parameters, more insight into electrode placement strategies, and alternative targets.

There are several aspects that currently complicate the process of developing a better understanding of the factors leading to favorable outcomes. Firstly, dystonia remains a rare disorder, and the number of patients undergoing treatment with DBS is relatively small. On top of that, dystonia is a very heterogenous movement disorder that presents in a multitude of ways. Recent studies show evidence that partially separate brain networks might be involved in various dystonia types, with each type responding best to a different electrode stimulation site [9], adding a new layer of complexity to predicting treatment outcomes.

Moreover, there is not a single motor outcome measure that suffices for all patients to capture the effect of DBS accurately. A previous paper showed that functional improvements as measured by the Canadian Occupational Performance Measure (COPM) were seen in the absence of motor changes as measured by the Burke-Fahn-Marsden Dystonia Rating Scale (BFMDRS) [10]. Moreover, with the continuously expanding understanding of dystonia in scientific literature, it is important to adapt our outcome measures accordingly. Specifically, the increasing evidence demonstrating the importance of non-motor symptoms and their impact on quality of life of dystonia patients. Currently, the effect of DBS on these symptoms has not been extensively studied.

Within the Netherlands, DBS for dystonia is generally performed in large tertiary centers that all use their own protocols for patient selection and measurement scales for assessing symptom severity. On a national level, about 30 dystonia patients undergo treatment with DBS annually [11]. Grouping and analysis of data of this already limited number of patients is complicated by these differing preoperative screening, selection and follow-up frameworks. To gain more insight into important factors contributing to better results and improve DBS there is a need to develop consensus on treatment outcomes, homogenous measures in initial screening, patient selection and follow up.

As a first step in solving this issue, we performed a multicenter Delphi study on a national level among Dutch health care providers involved in DBS for dystonia to acquire consensus on a set of outcome measures, which will support more consistent documentation. This establishes a foundation for a more uniform approach to patient selection and follow-up for DBS for dystonia in the Netherlands.

## Methods

For this study, a modified version of the Delphi technique was applied among Dutch experts in the field of DBS for dystonia. This is a method with an iterative approach using questionnaires to reach group consensus [12]. A priori a decision was made on the following study design: two rounds of Delphi questionnaires followed by a consensus meeting as a closing criterion. This process was performed separately for healthcare professionals involved in DBS care, for adults and children. Approval of the local ethics committee was sought for this study but deemed not necessary.

### Expert selection

Healthcare providers of all centers involved in DBS for dystonia care in the Netherlands were approached by email to participate. The minimal requirement for centers to participate in the Delphi process was that at least 1 neurologist and 1 neurosurgeon from the same center were available to take part in the Delphi process. Apart from this requirement, all other care providers that took care in their practice of dystonia patients (i.e. physiatrists, psychiatrists, neuropsychologists, physical therapists, occupational therapists, speech therapists, nurse practitioners, and physician assistants) undergoing DBS surgery and were involved in either preoperative screening, follow-up or both were invited to participate.

### Inventory survey

Firstly, a preliminary round consisting of a structured inventory questionnaire was held in all participating centers for DBS in both children and adults separately. The goal of this round was to evaluate the current modus operandi in each center before the two Delphi rounds. Participants were allowed to fill in the questionnaire individually or as a group from the same center. The following subjects were assessed: motor and non-motor measurement instruments used at present, selection criteria, follow-up design, involved health care providers, available options for DBS systems (sensing, steering, (non-)rechargeable systems), pre- and intraoperative imaging modalities, initial programming settings, other logistics surrounding the DBS surgery. The survey ended with an open question to add any additional aspects the experts considered important as to ensure no aspects were overlooked. The results of this survey were analyzed qualitatively and used as input for the design of the Delphi questionnaires. Inventory survey data were collected and managed using REDCap [13]. Reminders were sent out several times to increase the response rate.

### Round 1

The first Delphi questionnaire was designed in Google Forms (Google LLC) and developed based on three sources: 1.) the outcomes of the inventory survey among the experts, 2.) clinical experience from the study team and participating centers, and 3.) a targeted literature search on available rating scales for dystonia. Collected subjects were grouped into several domains (e.g. motor outcomes, non-motor outcomes, care pathways, selection criteria, follow-up, etc.). Rather than applying predefined inclusion criteria, the study team selected statements based on clinical relevance, and potential benefit from expert consensus. Statements were iteratively refined by the study team for clarity and phrased to be tailored to a multidisciplinary panel. All experts that participated in the inventory survey were also invited for this first Delphi round. Participants were asked to rate the importance of several items related to DBS for dystonia on a five-point Likert scale (1: unimportant – 5: very important). It was decided to introduce a midpoint response option after careful consideration of the literature [14]. Additionally, for every question a ‘no opinion/this falls beyond the scope of my specialty’ option was provided, because of the diverse professional backgrounds of the participants. Respondents that chose this answer were not included in the consensus percentage/importance score calculations for that item. The cut-off point for consensus was set prior to the first round at a median score of ≥ 4 on the level of importance Likert scale, and 70% of the total number of participants scoring this item 4 or higher. Once these thresholds were reached for an item, it was not reiterated in the questionnaire of the next round. Therefore, the stability criterion (consensus remains over multiple rounds) as described by Nasa et al. was not used as a closing criterion in this study [15]. After each question, participants were given the opportunity to provide comments or express their rationale for their given answer. In addition to the importance rating questions, participants were asked an open-ended question about their opinion on the use of Micro-Electrode Recordings (MER) and their preference of intra-operative imaging to confirm electrode placement. Additionally, one question was asked on their preferred post-operative follow-up moments. Regarding non-motor aspects, it was decided to focus this Delphi process on consensus about assessing these symptoms in general. The specifics on which scales to use are beyond the scope of this study, and will be determined at a later stage by a focus group.

### Round 2

The second round questionnaire was also designed in Google Forms. Only experts that participated in round 1 were invited to take part, to maintain the iterative process. During this round, the items that did not reach the 70% importance threshold during the first round were presented again, or in a slightly changed form based on comments from the first round. In addition, the individual scores of the participant and a summarized response on group level from the previous round were provided. Participants were asked if they wanted to change their response based on the presented group level results from round 1. New items that were proposed in the comment sections of the first round questionnaire were also iterated this round.

### Consensus meeting

The finishing round of the Delphi process consisted of an online consensus meeting. The minimal requirement for participation was that at least one neurologist and one neurosurgeon from each center participated, but all providers involved in DBS for dystonia care were invited (physiatrists, psychiatrists, neuropsychologists, physical therapists, occupational therapists, speech therapists, nurse practitioners, and physician assistants). The items from the second round that did not reach the 70% importance threshold were rephrased into statements and presented to the participants. The statements were phrased as propositions to either include or exclude the item from the final set of outcome measures. If an item reached an importance score of 50% or less in prior rounds it was phrased as exclusion statement, and between 50–70% as inclusion statement. To facilitate the voting process during the consensus meeting, a dichotomous answer voting scale with the options ‘agree’ or ‘disagree’ was used. The acceptance threshold for the statements was maintained at 70 percent. Voting occurred anonymously via de polling system of Microsoft Teams (Microsoft Cooperation, Redmond, WA, USA). The goal of this meeting was to solidify the expressed opinions in the previous rounds, to reiterate the new items that were expressed in the comment sections of the previous questionnaires, and to allow for discussion amongst participants.

After the consensus meeting a document summarizing the outcomes was sent to all participants for further development and refinement for practical clinical implementation.

### Statistical analysis

In the various Delphi rounds descriptive statistics were determined using Microsoft Excel (Microsoft Cooperation, Redmond, WA, USA). Median scores were calculated as a measure of central tendency as well as the percentage of respondents scoring the item as 4 (relatively important) or higher during the first two rounds, and during the consensus meeting as an agreement percentage of 70% or higher.

## Results

All rounds were conducted twice, once for DBS in adults and once for DBS in children. Some experts involved in clinical practice for adult and pediatric DBS participated in both Delphi processes.

### Preliminary round

In the preliminary inventory round for adult DBS 26 healthcare providers from 5 centers (Maastricht University Medical Center (MUMC+), HagaZiekenhuis, Amsterdam University Medical Center (Amsterdam UMC), University Medical Center Groningen (UMCG), Elizabeth TweestedenZiekenhuis Tilburg (ETZ)) participated, and for pediatric DBS 12 healthcare providers ((pediatric) neurologist n = 4, (pediatric) neurosurgeon n = 4, nurse practitioner n = 3, pediatric occupational therapist n = 1) from 3 centers. The most important differences between centers were found in the used measurement instruments to determine severity of symptoms, the involved healthcare providers in preoperative screening and follow-up, whether non-motor symptoms were assessed, and the frequency of protocolled follow-up moments.

### Delphi process participating centers

All centers (n = 5) in the Netherlands performing DBS for dystonia were approached to participate in this Delphi process. One center decided to not participate due to only sporadically performing DBS for dystonia. All other centers that were approached did participate. For the adult DBS process participating centers were: Amsterdam UMC, MUMC+, HagaZiekenhuis, UMCG, and for the pediatric DBS process were: Amsterdam UMC, MUMC+, and UMCG.

### Round 1

The first Delphi round was held from November 2023 until February 2024. For the adult Delphi process 27 health care providers from 5 centers were approached by email, of which 18 (67%; neurologist (n = 6), neurosurgeon (n = 6), neuropsychologist (n = 2), physiotherapist (n = 1), physiatrist (n = 1), speech therapist (n =1), and nurse practitioner (n = 1)) participated in this round. For the Pediatric DBS Delphi process 17 participants from 3 centers were approached, of which 11 participated in the first survey (65%; (pediatric) neurologist (n = 3), pediatric neurologist in training (n = 1), (pediatric) neurosurgeon (n = 3), (pediatric) physiatrist (n = 2), (pediatric) occupational therapist (n = 1), (pediatric) neuropsychologist (n = 1)).

Participants reached consensus on the importance of 37/55 items ((67%), Delphi for adult patients) and 40/54 items ((74%), Delphi for pediatric patients), see Tables 1, 2, 3, 4, 5, 6, 7 for all items and consensus percentages. There was unanimous agreement on the importance of scoring both motor and non-motor symptoms using measurement instruments in adults and children. For motor symptoms in adults, there was consensus on the use of the BFMDRS for generalized, cervical, other focal, segmental or multifocal forms of dystonia, and myoclonus dystonia. Regarding other domains to evaluate there was consensus on assessing pain, depression, anxiety, cognitive functioning, quality of life, participation, patient satisfaction, and activities of daily living during the pre-operative screening phase. Concerning selection criteria, the panel agreed on taking into consideration the number of prior medication trials, psychiatric comorbidity, cognitive impairments, dystonia etiology, experienced limitations in daily life, quality of life and (non-)motor symptom severity. For children there was agreement on considering psychiatric comorbidity, dystonia etiology, limitations in daily life, quality of life, and severity of (non-)motor symptoms during pre-operative screening. For both adults and children, consensus was reached on several DBS-related aspects, such as patient choice of IPG, documentation of device specification, and the presence of a neurologist in the operating room during awake DBS surgery. For follow-up there was consensus in both groups on a structured and protocolled post-operative follow-up at set intervals, assessment of both motor and non-motor symptoms during follow-up, and video recording of motor symptoms during follow-up.

**Table 1:** **Statements regarding DBS in adults that reached consensus after the final stage**. Of note, only the statements that reached consensus on including an item are displayed in this table. All other statements that also reached consensus on excluding items can be found in Tables 3, 4, 5, 6, 7.


CONSENSUS STATEMENTS

***Motor symptoms***

1. Scoring the severity of dystonic motor symptoms with a (validated) measurement instrument

2. Video recording of the dystonic motor symptoms before surgery

3. Video recording according to a set protocol

4. Motor goals set by the patient he/she hopes to achieve with DBS treatment

***Motor scales***

5. Generalized dystonia – BFMDRS

6. Cervical dystonia – BFMDRS, TWSTRS, Tsui

7. Other focal, segmental or multifocal dystonia – BFMDRS

8. Myoclonus dystonia – BFMDRS, UMRS

***Non-motor symptoms***

9. Determine severity of non-motor symptoms with a (validated) measurement instrument

10. Preoperative assessment of cognitive status by a neuropsychologist

11. By default carry out a brief neuropsychological exam, and when indicated a full neuropsychological exam

12. Non-motor goals set by the patient he/she hopes to achieve with DBS treatment

***Non-motor symptoms/domains to assess by a (validated) questionnaire***

13. Pain

14. Depression

15. Anxiety

16. Cognitive functioning

17. Quality of life

***Involved care providers***

18. Involve an occupational therapist when considered necessary during the preoperative screening

19. Involve a speech therapist when considered necessary during the preoperative screening

20. Involve a physiatrist when considered necessary during the preoperative screening

***Other aspects of importance to assess***

21. Participation

22. Patient satisfaction

23. Activities of daily living

***Selection criteria***

24. Minimal number of drug trials

25. Psychiatric comorbidity

26. Cognitive impairments

27. Etiology of dystonia

28. Experienced limitations in daily life

29. Quality of life

30. Severity of non-motor symptoms

31. Severity of motor symptoms

***DBS related aspects***

32. Offer the option to patients to choose between rechargeable and non-rechargeable IPGs

33. The presence of a neurologist in the operating room when there is awake DBS surgery

34. Document IPG specification in the electronic patient file

***Follow-up***

35. Structured protocolled post-operative follow-up moments at set intervals

36. Assessment of motor symptoms during follow-up

37. Assessment of non-motor symptoms during follow-up

38. Video recording of dystonic motor symptoms during follow-up

39. Document whether the use of pre-DBS botulinum toxin treatment is continued after DBS implantation

40. Document whether medication use could be reduced or ceased after DBS implantation

41. A specific scale to assess the severity of tremor

42. Record side effects and complications during the course of DBS treatment

43. In adults a protocolled follow-up moment between 3–5 years post-surgery


**Table 2:** **Statements regarding pediatric DBS that reached consensus at the final stage**. Of note, only the statements that reached consensus on including an item are displayed in this table. All other statements that also reached consensus on excluding items can be found in Tables 3, 4, 5, 6, 7.


CONSENSUS STATEMENTS

***Motor symptoms***

1. Scoring the severity of dystonic motor symptoms with a (validated) measurement instrument

2. Video recording of the dystonic motor symptoms before surgery

3. Video recording according to a set protocol

4. Motor goals set by the patient he/she hopes to achieve with DBS treatment

5. Use a scale to measure severity of spasticity

6. Determine motor goals not only on functional level, but also on skill level

***Motor scales***

7. Generalized dystonia – BFMDRS, BADS

8. Cerebral palsy – BFMDRS, BADS

9. Other focal, segmental or multifocal dystonia – BFMDRS

10. Myoclonus dystonia – BFMDRS, UMRS

***Non-motor symptoms***

11. Determine severity of non-motor symptoms with a (validated) measurement instrument

12. Preoperative assessment of cognitive status by a neuropsychologist

13. Non-motor goals set by the patient he/she hopes to achieve with DBS treatment

***Non-motor symptoms to assess by a (validated) questionnaire***

14. Pain

15. Depression

16. Anxiety

17. Insomnia

18. Cognitive functioning

19. Quality of life

***Involved care providers***

20. Standard involvement of occupational therapist during preoperative screening

21. Standard involvement of speech therapist during preoperative screening

22. Standard involvement of physiatrist during preoperative screening

23. Standard involvement of physical therapist during preoperative screening

***Other aspects of importance to assess***

24. Functioning at school

25. Patient/Parent defined goals

26. Patient satisfaction

27. Parent/caretaker satisfaction

28. Activities of daily living

***Selection criteria***

29. Psychiatric comorbidity

30. Etiology of dystonia

31. Experienced limitations in daily life

32. Quality of life

33. Severity of non-motor symptoms

34. Severity of motor symptoms

***DBS related aspects***

35. Offer the option to patients to choose between rechargeable and non-rechargeable IPGs

36. The presence of a neurologist in the operating room when there is awake DBS surgery

37. Document IPG specification in the electronic patient file

***Follow-up***

38. Structured protocolled post-operative follow-up moments at set intervals

39. Assessment of motor symptoms during follow-up

40. Assessment of non-motor symptoms during follow-up

41. Video recording of dystonic motor symptoms during follow-up

42. Document whether the use of pre-DBS botulinum toxin treatment is continued

43. Document whether medication use could be reduced or ceased after DBS implantation

44. Record side effects and complications during the course of DBS treatment

45. In children after 1 year protocolled follow-up

46. In children at least one other protocolled follow-up moment besides the 1 year follow-up

47. In children after 3 years protocolled follow-up

48. In children after 5 years protocolled follow-up


**Table 3:** Items in the questionnaires of the first two rounds of the adult DBS Delphi process and their respective consensus percentages.


STATEMENTS	ROUND 1*	ROUND 2*

***Motor symptoms***		

Scoring the severity of dystonic motor symptoms with a (validated) measurement instrument	100%	

Video recording of the dystonic motor symptoms before surgery	89%	

Video recording according to a set protocol	89%	

Motor goals set by the patient he/she hopes to achieve with DBS treatment	94%	

***Non-motor symptoms***		

Determine severity of non-motor symptoms with a (validated) measurement instrument	100%	

Preoperative assessment of cognitive status by a neuropsychologist	72%	

**Standard involvement of a physiotherapist during preoperative screening**	**56%**	– Standard involvement: 58% – No involvement: 17% – When indicated: 25%

**Standard involvement of an occupational therapist during preoperative screening**	**44%**	– Standard involvement: 33% – No involvement: 17% – When indicated: 50%

**Standard involvement of a speech therapist during preoperative screening**	**33%**	– No involvement: 8% – When indicated: 92%

**Standard involvement of a physiatrist during preoperative screening**	**33%**	– Standard involvement: 33% – No involvement: 8% – When indicated: 58%

Non-motor goals set by the patient he/she hopes to achieve with DBS treatment	89%	

***Importance of the following aspects being assessed with a questionnaire:***		

Pain	100%	

**Fatigue**	**56%**	**58%**

**Insomnia**	**56%**	**42%**

Depression	100%	

Anxiety	94%	

Cognitive functioning	78%	

Quality of life	100%	

***Other aspects***		

Participation	76%	

**Mobility/Range of motion**	**61%**	**67%**

Patient satisfaction	100%	

Activities of daily living	100%	

***Selection criteria***		

**Minimum age**	**13%**	**18%**

**Maximum age**	**25%**	**27%**

Minimal number of drug trials	77%	

Psychiatric comorbidity	88%	

Cognitive impairments	71%	

**Travel distance to the hospital**	**6%**	**8%**

Etiology of dystonia	81%	

**Disease duration**	**27%**	**42%**

Experienced limitations in daily life	94%	

Quality of life	89%	

Severity of non-motor symptoms	83%	

Severity of motor symptoms	100%	

***DBS related aspects***		

Offer the option to patients to choose between rechargeable and non-rechargeable IPGs	71%	

**Offer the option to patients to choose for sensing and/or steering systems**	**15%**	**0%**

The presence of a neurologist in the operating room when there is awake DBS surgery	80%	

Document IPG specification in the electronic patient file	100%	

***Follow-up***		

Structured protocolled post-operative follow-up moments at set intervals	94%	

Assessment of motor symptoms during follow-up	100%	

Assessment of non-motor symptoms during follow-up	94%	

Video recording of dystonic motor symptoms during follow-up	89%	

***New items added during round 2***		

Document whether the use of pre-DBS botulinum toxin treatment is continued after DBS implantation		100%

Document whether medication use could be reduced or ceased after DBS implantation		91%

A specific scale to assess the severity of tremor		91%

**Use a brief neuropsychological exam by default to assess cognition preoperatively, use a full neuropsychological exam when indicated**		**42%**


*Note: All items without consensus in bold.

**Table 4:** Consensus percentages during the first two rounds of the adult DBS Delphi process on motor symptom measurement instruments. Scales validated in literature for: ^1^Dystonia [19], ^2^Dystonia [25], ^3^Dystonia [25], ^4^Cervical dystonia [20,21], ^5^Cervical dystonia [20,21,22], ^6^Myoclonus dystonia [23,24], myoclonus [24].


MOTOR MEASUREMENT INSTRUMENTS	ROUND 1*	ROUND 2*

***Generalized dystonia***		

BFMDRS^1^	90%	

**UDRS** ^2^	**38%**	**0%**

**GDRS** ^3^	**25%**	**17%**

***Cervical dystonia***		

BFMDRS	73%	

**UDRS**	**22%**	**0%**

**GDRS**	**22%**	**0%**

TWSTRS^4^	100%	

Tsui score^5^	89%	

***Other focal, segmental or multifocal dystonia***		

BFMDRS	91%	

**UDRS**	**50%**	**17%**

**GDRS**	**50%**	**17%**

***Myoclonus dystonia***		

BFMDRS	89%	

UMRS^6^	88%	


*Note: All items without consensus in bold.

**Table 5:** Items in the questionnaires of the first two rounds of the pediatric DBS Delphi process and their respective consensus percentages.


STATEMENTS	ROUND 1*	ROUND 2*

***Motor symptoms***		

Scoring the severity of dystonic motor symptoms with a (validated) measurement instrument	100%	

Video recording of the dystonic motor symptoms before surgery	91%	

Video recording according to a set protocol	100%	

Motor goals set by the patient he/she hopes to achieve with DBS treatment	100%	

***Non-motor symptoms***		

Determine severity of non-motor symptoms with a (validated) measurement instrument	100%	

Assessment of cognitive status by a neuropsychologist	82%	

**Standard involvement of physiotherapist during preoperative screening**	64%*	– Standard involvement: 70% – When indicated: 30%

Standard involvement of occupational therapist during preoperative screening	82%	

Standard involvement of speech therapist during preoperative screening	82%	

Standard involvement of physiatrist during preoperative screening	82%	

Non-motor goals set by the patient he/she hopes to achieve with DBS treatment	91%	

***Importance of the following aspects being assessed with a questionnaire:***		

Pain	100%	

**Fatigue**	**50%**	**50%**

Insomnia	80%	

Depression	90%	

Anxiety	90%	

Cognitive functioning	80%	

Quality of life	100%	

***Other aspects***		

Functioning at school	100%	

**Mobility/Range of Motion**	**64%**	**50%**

Patient/Parent defined goals	100%	

Patient satisfaction	100%	

Parent/caretaker satisfaction	100%	

Activities of daily living	100%	

***Selection criteria***		

**Minimum age**	**56%**	**56%**

**Minimal number of drug trials**	**57%**	**50%**

Psychiatric comorbidity	90%	

**Cognitive impairments**	**40%**	**40%**

**Travel distance to the hospital**	**0%**	**0%**

Etiology of dystonia	80%	

**Disease duration**	**30%**	**30%**

Experienced limitations in daily life	100%	

Quality of life	91%	

Severity of non-motor symptoms	82%	

Severity of motor symptoms	100%	

***DBS related aspects***		

Offer the option to patients to choose between rechargeable and non-rechargeable IPGs	71%	

**Offer the option to patients to choose for sensing and/or steering systems**	**17%**	**0%**

The presence of a neurologist in the operating room when there is awake DBS surgery	75%	

Document IPG specification in the electronic patient file	100%	

***Follow-up***		

Structured protocolled post-operative follow-up moments at set intervals	100%	

Assessment of motor symptoms during follow-up	91%	

Assessment of non-motor symptoms during follow-up	82%	

Video recording of dystonic motor symptoms during follow-up	100%	

***New items added during round 2***		

Use a scale to measure severity of spasticity	–	78%

Record treatment goals of both parents and patients	–	100%

Record side effects during DBS treatment	–	100%

Record complications during DBS treatment	–	100%

Document whether the use of pre-DBS botulinum toxin treatment is continued	–	78%

Document whether medication use could be reduced or ceased after DBS implantation	–	78%

Determine motor goals not only on functional level, but also on skill level	–	89%


*Note: All items without consensus in bold.

**Table 6:** Consensus percentages during the first two rounds of the pediatric DBS Delphi process on motor symptom measurement instruments. Scales validated in literature for: ^1^Tested in dystonia in older children, but beware of age dependency in young children [26,27,28], ^2^Dystonia in cerebral palsy [27,29], ^3^Inherited or idiopathic dystonia, dystonia and choreoathetosis in dyskinetic cerebral palsy [30,31], ^4^Cervical dystonia in adults [20,21], ^5^Myoclonus dystonia [23,24], myoclonus [24].


MOTOR MEASUREMENT INSTRUMENTS	ROUND 1*	ROUND 2*

***Generalized dystonia***		

BFMDRS^1^	100%	

BADS^2^	80%	

***Cerebral palsy***		

BFMDRS	100%	

BADS	80%	

**DIS** ^3^	**50%**	**43%**

***Other focal, segmental or multifocal dystonia***		

BFMDRS	86%	

**BADS**	**60%**	**57%**

**TWSTRS** ^4^	**57%**	**29%**

***Myoclonus dystonia***		

BFMDRS	83%	

**BADS**	**50%**	**40%**

**UMRS** ^5^	**67%**	**50%**


*Note: All items without consensus in bold.

**Table 7:** Consensus percentages on the consensus meeting items of the adult and pediatric Delphi process


CONSENSUS MEETING STATEMENTS ADULT DBS	AGREEMENT %*

1. The UDRS and GDRS fall outside the scope of the set of agreed outcome measures in generalized dystonia for adults	100%

2. The UDRS and GDRS fall outside the scope of the set of agreed outcome measures in cervical dystonia for adults	100%

3. The UDRS and GDRS fall outside the scope of the set of agreed outcome measures in focal, segmental or multifocal dystonia for adults	100%

4. The UDRS and GDRS fall outside the scope of the set of agreed outcome measures in myoclonus dystonia for adults	100%

5. No requirement to use a measurement scale for fatigue and sleep problems, but to discuss this during history taking for adults	**78%**

**6. By default involve a physical therapist in the preoperative screening phase for adults**	**65%**

7. Involve an occupational therapist when deemed necessary during the preoperative screening for adults	70%

8. Involve a physiatrist when deemed necessary during the preoperative screening for adults	70%

**9. Mobility/Range of motion is not checked by default during the preoperative screening and follow-up for adults**	**68%**

10. Concerning selection criteria for adults, to not necessarily take into account minimal age, maximal age, travel distance to the hospital	100%

11. In adults a protocolled follow-up moment between 3–5 years post-surgery	75%

12. In adults by default carry out a brief neuropsychological exam, and when indicated a full neuropsychological exam	93%

13. Record side effects and complications during the course of DBS treatment	100%

***Statements regarding both adults and pediatric DBS***	

1. Both for adults and children not the option to by default choose sensing/steering electrodes	100%

2. Use of micro-electrode recording (MER) only when MRI is not possible*	–

**CONSENSUS MEETING STATEMENTS PEDIATRIC DBS**	**AGREEMENT %**

1. The DIS falls outside the scope of the set of agreed outcome measures in cerebral paresis for children	100%

2. The BADS and TWSTRS fall outside the scope of the set of agreed outcome measures in focal, segmental or multifocal dystonia for children	100%

3. The BADS falls outside the scope of the set of agreed outcome measures in myoclonus dystonia for children	100%

4. Standard use of the UMRS in children with myoclonus dystonia	93%

5. No requirement to use a measurement scale for fatigue in children, but to discuss this during history taking	71%

**6. Mobility/Range of motion not assessed by default during the preoperative screening and follow-up for children**	**68%**

7. Concerning selection criteria for children to not necessarily take into account minimal age, number of drug trials, travel distance to the hospital	94%

8. Concerning selection criteria for children to not necessarily take into account travel distance and disease duration	100%

9. In children by default a protocolled follow-up moment at 1 year post-surgery	100%

**10. In children by default a protocolled follow-up at 1 and 5 years post-surgery**	**62%**

11. In children at least one other protocolled follow-up moment besides the 1 year follow-up	88%

12. In children after 3 years protocolled follow-up	97%

13. In children after 5 years protocolled follow-up	82%


*Note: All items without consensus in bold.

In terms of the involved care providers during the pre-operative screening there was no consensus for adults on default involvement of a physical, occupational, speech therapist, or physiatrist. For children there was consensus on the default involvement of an occupational and speech therapist, and physiatrist.

Regarding the open questions on imaging, and follow up, the responses were as follows: the preference for intraoperative imaging to confirm electrode placement was Computed Tomography (CT) for both adult (n = 8/9) and pediatric DBS (n = 2/2). For the preferred follow-up moments for a full multidisciplinary work up there was no unanimous answer. Proposed follow-up moments varied widely in terms of the number (range adult DBS: 1 – 8 FU moments, pediatric DBS: 1 FU moment – annually until adulthood) and the time interval in between.

### Round 2

The second Delphi rounds were held within the first three weeks of March 2024. The items of the first round that did not reach consensus were reiterated during this second round. For this round respectively 12 participants (adult Delphi process: (neurologist (n = 5), neurosurgeon (n = 3), neuropsychologist (n = 2), physical therapist (n = 1), and nurse practitioner (n = 1)) and 10 participants (pediatric Delphi process: (pediatric) neurologist (n = 3), pediatric neurologist in training (n = 1), (pediatric) neurosurgeon (n = 2), (pediatric) physiatrist (n = 2), (pediatric) occupational therapist (n = 1), (pediatric) neuropsychologist (n = 1)) remained. None of these reiterated items reached consensus. For the newly proposed items 3/4 and 7/7 reached consensus, respectively in the adult and pediatric Delphi processes. For adults these items were: to document continuation of botulinum toxin treatment after DBS, reducing or ceasing medication use after DBS, a specific scale to assess tremor severity, if present. For children these items were similar except for the tremor scale, and instead included use of a scale for spasticity, recording treatment goals of both parents and patients, complications, and side effects, and to determine motor goals on both functional and skill level.

In the previous round there was no consensus to routinely involve a physical therapist, occupational therapist, speech therapist and physiatrist during preoperative screening for adults. Based on the comments it was decided to rephrase the response options during the next round to assess whether experts had a preference to ‘not involve at all’, ‘always involve during preoperative screening’, or ‘involve based on indication’ to assess the general opinion in a more nuanced manner. Regarding physical therapists, a small majority of the panel voted to involve by default during the preoperative screening for adults, while for occupational, speech therapists and physiatrists, the opinion was to involve only when indicated. Because none of the majority percentages reached the consensus threshold, the items were re-iterated in the next round. For pediatric DBS the general opinion was to involve a physical therapist by default during preoperative screening.

### Round 3 – Consensus meeting

The consensus meeting was held at the end of March 2024. Due to scheduling issues, having participated in the prior rounds of questionnaires was not a necessity to take part in the consensus meeting. In total 21 participants took part: (pediatric) neurologist: 7, pediatric neurologist in training: 1, (pediatric) neurosurgeon: 5, (pediatric) physiatrist: 3, (pediatric) neuropsychologist: 1, speech therapist: 1, physical therapist: 1, nurse practitioner: 1, (pediatric) occupational therapist: 1. Two participants that did not take part in a previous round joined the consensus meeting. Reasons for this were to substitute for a participant from the same center that was unavailable to attend and because a participant was unable to join during the earlier questionnaires. The remaining 19 (adult DBS) and 14 (pediatric DBS) items that did not reach consensus during round 2 were reformulated into 28 new statements. After voting in this round 11/13 statements (adult DBS), 1/2 statements (both adult and pediatric DBS), and 11/13 statements (pediatric DBS) reached consensus (Table 7).

## Discussion

In this study we performed two Delphi processes among experts in the field of DBS for dystonia in adults and children in the Netherlands. The purpose of our study was to establish a minimum set of uniform outcome measures regarding DBS for dystonia care to introduce more uniformity in the process of preoperative screening and follow-up in Dutch centers. The presented results depict the reached consensus among experts in this field in the Netherlands.

To our knowledge, this is the first study to determine consensus on this topic. In prior research, the Delphi method has been used comprehensively to establish consensus on outcome measures and has proven to be an effective and reliable method in doing so. The design of this study has been in line with the recommendations proposed in literature, therefore the results are founded on a solid study design [16].

As expected, there was consensus on the importance of videotaping the dystonic motor symptoms before and during treatment with DBS by a standard protocol and the use of validated rating scales for assessing motor symptom severity among the experts in both adult and pediatric DBS care. This allows for comparison before and after treatment within the same patient and between different patients, and is crucial in determining treatment effect. A key observation in this study was the consensus regarding the importance of recording the severity of various non-motor symptoms with a measurement instrument. Prior research has indicated that the associated non-motor symptoms of dystonia are a core feature of the disease, and can pose a tremendous burden on quality of life. So far, the effect of DBS on non-motor symptoms has only been studied retrospectively and in a small prospective study [17,18]. Standard assessment of these symptoms as part of the preoperative screening and follow-up enables us to develop a more complete understanding of the effects of DBS on both the motor and non-motor aspects of dystonia. Other aspects the expert panel agreed on were the importance of assessing participation, quality of life, patient and parent satisfaction, and activities of daily living, further emphasizing that a more holistic approach is desired.

Interestingly, for adults there was no consensus on the standard involvement of allied healthcare professionals such as physical therapists, occupational therapists, speech therapists and physiatrists during the preoperative screening phase. There was, however, agreement on the involvement of an occupational therapist, speech therapist and physiatrist on a case by case basis when deemed necessary. The lack of specificity of the consensus statements regarding the involvement of allied care professionals illustrates the diversity of clinical practice across centers. The phrasing ‘when deemed necessary’ was chosen as a compromise to tailor to differing views, ranging from routine involvement to referral based on clinical judgement. This differed from the pediatric Delphi process, as there was agreement to by default involve an occupational therapist, speech therapist, physical therapist, and physiatrist during the preoperative screening phase. Conceivably, this might stem from the fact that childhood dystonia can have a profound effect on normal motor development, which makes the multidisciplinary approach more of a core necessity in pediatric DBS. Our results show that a multidisciplinary approach is not standard practice in adult dystonia DBS treatment everywhere yet. The variability in responses shows the need for future research to define clearer referral criteria and establish best practices regarding multidisciplinary involvement for DBS in dystonia.

This study has various strengths. Firstly, our expert panel consisted of professionals with varying backgrounds (neurologists, neurosurgeons, physiatrists, physical therapists, occupational therapists, neuropsychologists), all with ample experience in DBS for dystonia in daily practice. This allowed for opinions from many angles to be expressed and to reach a consensus among the many disciplines that are involved in DBS for dystonia, recognizing the importance of the multidisciplinary nature of this type of treatment. On top of that, there was broad representation of clinical practice in this field in the Netherlands, as all centers that regularly perform DBS for dystonia were included in this Delphi process. This ensures that consensus statements reflect the entire spectrum of national expertise on this subject. This broad participation enhances the applicability of the findings within the Dutch healthcare system and paves the way for future collaborative studies. The group size remained appropriate throughout the process, forming an accurate representation of the DBS for dystonia field in the Netherlands. Additionally, the anonymity during this process, even during the voting process in the consensus meeting, excluded the introduction of certain types of bias. The advantage of a consensus meeting as a final round was that it allowed for discussion, while still upholding anonymity in the voting process.

A limitation of this study is that only Dutch experts were included. Therefore, the design of the questionnaire was aimed at the infrastructure of the Dutch healthcare system and might not necessarily be applicable to systems in other countries. Secondly, while the study team recognizes the importance of discussion on preferred stimulation targets for dystonia, this study did not go into depth about this subject. As its intention was to ensure relevance to a broad range of care providers, the study team wanted to prevent discussion becoming too focused on surgical outcomes rather than patient-centered outcomes. Thirdly, the loss of participant adherence across Delphi rounds is a limitation, with fewer experts completing the second round compared to the first. Efforts were made to minimize this risk by setting the number of rounds before the start of the Delphi study, and by scheduling well ahead of each round. It was anticipated up front that this participant population would present challenges in terms of availability, which was another reason for the current study design. A downside of this design is that more complex or controversial items can only be explored to a limited extend as these would require more rounds. Additionally, regarding the instruments mentioned in this study, it should be emphasized that inclusion reflects expert consensus rather than a strict evidence-based selection process.

Finally, the stability criterion as formulated by Nasa et al., which involves that consensus has to persist over multiple rounds, was not used as a closing criterion for this study [15]. This was decided to keep up the response rate and prevent questionnaire overwhelm. Therefore, while we observed convergence of responses between rounds, no conclusions can be drawn on response stability.

The results of this study address the lack of standardized outcomes and follow-up in DBS for dystonia. It provides a groundwork for a more uniform national approach and underscores the importance of multidisciplinary care in this field. Furthermore, the consensus reached in this Delphi study provides a foundation for future studies, as it allows for consistency in key aspects of DBS for dystonia. Therefore, facilitating research with comparable parameters when this consensus is adhered to. On a national level this study has led to an overview document containing recommendations for clinical practice to help Dutch centers improve comparability of results and facilitating collaboration between centers. Moreover, currently the frameworks for a national DBS for dystonia database are being built.

While consensus was reached on most items, some areas, such as timing of post-operative follow-up moments, remain a subject of discussion. These unresolved topics highlight the need for an ongoing dialogue and ideally, international collaboration to define best practices in these areas. Future research should focus on further specification of the unresolved topics, and on the implementation of uniform measures on an international scale. Since the number of patients undergoing DBS are sparse, harmonizing outcome measures would stimulate merging of datasets. As a result, more insight can be obtained into what drives successful outcomes. Additionally, the measurement tools for the defined non-motor domains should be further specified.

## Conclusion

In this study, a modified Delphi process was used to reach consensus on a set of uniform outcome measures and to introduce more uniformity in the process of preoperative screening and follow-up regarding DBS for dystonia care in the Netherlands. Due to the rareness and heterogenous clinical picture that encompasses dystonia, studying the interplay between factors important for successful treatment remains difficult. The consensus in this Delphi study is a reflection of what is deemed, in a broad sense, ‘best practice’ among Dutch experts in the field, and provides a strong foundation for further collaboration and improvement of patient care within the field of DBS for dystonia.
